# Crystallization processes in a nonvibrating magnetic granular system with short range repulsive interaction

**DOI:** 10.1038/s41598-019-40062-2

**Published:** 2019-03-05

**Authors:** M. J. Sánchez-Miranda, J. L. Carrillo-Estrada, F. Donado

**Affiliations:** 1 Instituto de Física “Luis Rivera Terrazas”, Benemérita Universidad Autónoma de Puebla, Puebla, Puebla, Mexico; 2 Instituto de Ciencias Básicas e Ingeniería de la Universidad Autónoma del Estado de Hidalgo-AAMF, Pachuca, 42184 Pachuca de Soto, Mexico

## Abstract

Applying an unsteady magnetic field to a 2D nonvibrating magnetic granular system induces a random motion in the steel beads with characteristics analogous to that of molecules in a fluid. We investigate the structural characteristics of the solid-like structures generated by different quenching conditions. The applied field is generated by the superposition of a constant field and a collinear sinusoidal field. The system reaches a quasi steady state in which the effective temperature is proportional to the amplitude of the applied field. By reducing the effective temperature at different rates, different cooling rates are produced. A slight inclination of the surface allows us to investigate the effects of small particle concentration gradients. The formation of a wide and rich variety of condensed solid structures, from gel-like and glass-like structures up to crystalline structures, is observed and depends on the cooling rate. We focus our attention on the crystallization process and found this process to be a collective phenomenon. We discuss our results in terms of the measured time evolution of the mean squared displacement, the effective diffusion coefficient, and the radial distribution function.

## Introduction

When a liquid is cooled beyond a certain temperature, it transforms into a solid body. If the cooling process happens quickly, the final structure will be glassy, and if the cooling process occurs slowly enough, the final molecular arrangement could be crystalline^1,2^. Crystal generation requires the formation of a nucleus that propitiates the aggregation process. For nucleation to be possible, the system must reach certain critical particle concentration and temperature values. In addition, the occurrence of a density fluctuation or the merger of an inhomogeneity is needed^3^. Thus, the formation of a nucleus is a process that occurs at non-equilibrium conditions. To be stable and to propitiate the aggregation process, a nucleus must reach a certain critical size as smaller clusters are destroyed by thermal fluctuations. Because it occurs at a molecular level and involves a small number of particles, the process of nucleus formation is not easily observed experimentally. The observation of the crystallization process in some giant molecules, such as globular proteins, allows us to obtain some insights into this phenomenon^4,5^. Certainly, there are some reports in the literature regarding numerical studies of nucleation and crystallization processes. Some of these studies specifically address the important issue of the influence of the crystalline or disordered nucleus structure on the crystallization process. On the other hand, it is well known that particle concentration also plays an important role in the formation of a nucleus and the ulterior aggregation process. The early stages of the formation of a crystal still have some fundamental unanswered questions. For instance, there has been some discussion on the various possibilities of aggregation leading to the formation of a nucleus. One of these possibilities is that at first, small clusters are formed due to concentration fluctuations, and then some of the clusters aggregate to form the nucleus. Another possibility is that individual particles aggregate one by one to form a small cluster and combine to form the nucleus. In both cases, some important questions emerge: does a particle join the nucleus in a position that is the minimum energy corresponding to a crystalline arrangement, or does a particle adhere to the small cluster in an unstable position and then evolve to an ordered configuration.

Therefore, a system that (1) is composed of a large number of macroscopic interacting particles whose dynamics mirrors the dynamics of a liquid and (2) that consequently allows the modelling, with particles visible by the naked eye, of a real-world microscopic system could provide valuable information to address some of the questions about the nucleation and crystallization processes raised above. With this motivation, colloidal systems have been previously used to model pattern formation processes^6–14^. Granular systems have also been used to investigate the dynamics of pattern formation in the solidification processes^15–21^. In these systems, it is then possible to induce solidification by changing the two most important variables that drive the solidification transition: effective temperature and particle concentration.

We have previously studied the conditions in which a nonvibrating granular magnetic system can be fluidized by magnetic interactions induced by the application of an unsteady magnetic field^15–18^. We demonstrated that one can control the effective temperature of the system by varying the intensity of the applied magnetic field. Of course, the system operates in a highly dissipative regime because the applied field makes particles roll randomly over the surface of the container and collide. A continuous energy input allows the system to reach a stationary condition. Under these conditions, the system behaviour fully exhibits the characteristics that define the Orstein-Uhlenbeck processes, where particles undergo Brownian-like motion^17^. We have used this system to study the glass transition^15^. We have shown that the measured particle velocity distribution is a Maxwell-Boltzmann type. We have measured static and dynamic structural characteristics of the system, such as the mean squared displacement, the radial distribution function, and the intermediate scattering function. We have observed that these quantities robustly describe the system behaviour as a granular model of a fluid. In those previous studies, there were no inhomogeneities coming from the magnetic field or the surface that were affecting the aggregation process. It is worth mentioning that we found no crystalline clusters, even when the system was exposed to very low cooling rates.

In this work, we have adapted our previous experimental setup by slightly tilting the particle container. For certain cooling rates, this leads to the formation of crystalline clusters and enables us to analyse in detail the role of the particle concentration in these pattern formation processes. Of course, we wish to take advantage of the fact that particle motion can be tracked with sufficient time and spatial resolution. Using this technique, we are able to observe that under adequate effective temperature and particle concentration conditions, crystalline pattern formation occurs. In the following, we first describe the system and then discuss the experiments we conducted at several cooling rates to explore the effect of effective temperature and particle concentration on the resulting pattern formation. We paid particular attention to clarifying whether, using this system with short-range repulsive interaction, it is possible to generate crystallization non-induced by commensurability with the confinement as this does in other systems^22^. Next, we discuss that as a result of different conditions of quenching, we obtained glassy gel and crystal-like structures. In some works, crystallization is studied starting from a solid state, and then, through an annealing process, by using shaking or shearing, crystallization is obtained^23,24^. In this work, no annealing was used to obtain crystallization. We analysed the dynamics of the system in terms of the mean square displacement (MSD) and the effective diffusion coefficient (D). The structural properties are discussed in terms of the radial distribution function, g(r), and the fast Fourier transform (FFT). Finally, we present some final comments.

## Results and Discussion

Our system consists of a granular 2D system, formed by a set of slightly magnetized steel spherical beads, under the action of an external variable magnetic field. The particles roll over the observation cell surface following the applied field. The complex interaction between the magnetic field and the particle magnetic moment makes the particle motion analogous to Brownian motion. By its design, this system behaves like a real 2D system with a short-range repulsive magnetic interaction. We start our experiments by setting the system with a homogeneous distribution and turning on the fields at the time t = 0, with proper amplitude to induce a high temperature. At this state, all particles perform diffusive motion and are able to explore the whole area of the observation cell. This physical situation can be held with no change for a very long time. Because of the slight tilt of the container, gravity at the lower part of the cell causes the particle concentration to be greater than at the upper region of the observation cell. The particle concentration is quantified by ${\varphi }_{2D}=N\pi {\sigma }^{2}\mathrm{/4}A$, where N is the average number of particles in the field of view with area A, and *σ* is the particle diameter. Obviously, the gradient of the particle concentration yields different effective diffusion coefficient values in different regions of the cell (R1 at the bottom, R2 in the middle, R3 at the top). One can see that the particle displacements are smaller at the bottom compared with the top.

When decreasing the amplitude of the applied field, the system starts a cooling process, and a very rich variety of physical phenomena become evident. One observes that in R1, particles become arrested, and this occurs much more frequently and more quickly than in R2 and even more than in R3. At the bottom of R1, small and unstable agglomerates start forming. At lower temperatures, aggregates become more stable and larger. In this stage, one may observe particles that are trapped momentaneously in nonstable structures. Because particles are frequently hit by their neighbours, if a particle is in a configuration with a deep enough local minimum energy, the particle remains there. If this is not the case, eventually the collisions could drive the particle to a position with a deeper energy minimum. Sometimes a particle can be pushed aside from the aggregate. If the cooling rate is large, the final configuration where particles become trapped is a disordered configuration, and this configuration obviously does not correspond to one of the deepest energy minima, namely, for this quenching, the system obtains a glassy arrested configuration. On the contrary, if the cooling is slow enough, one observes the formation of crystalline structures. Our experiment ends when no further changes are observed in the structure for an extended period of time.

**Figure 1:**
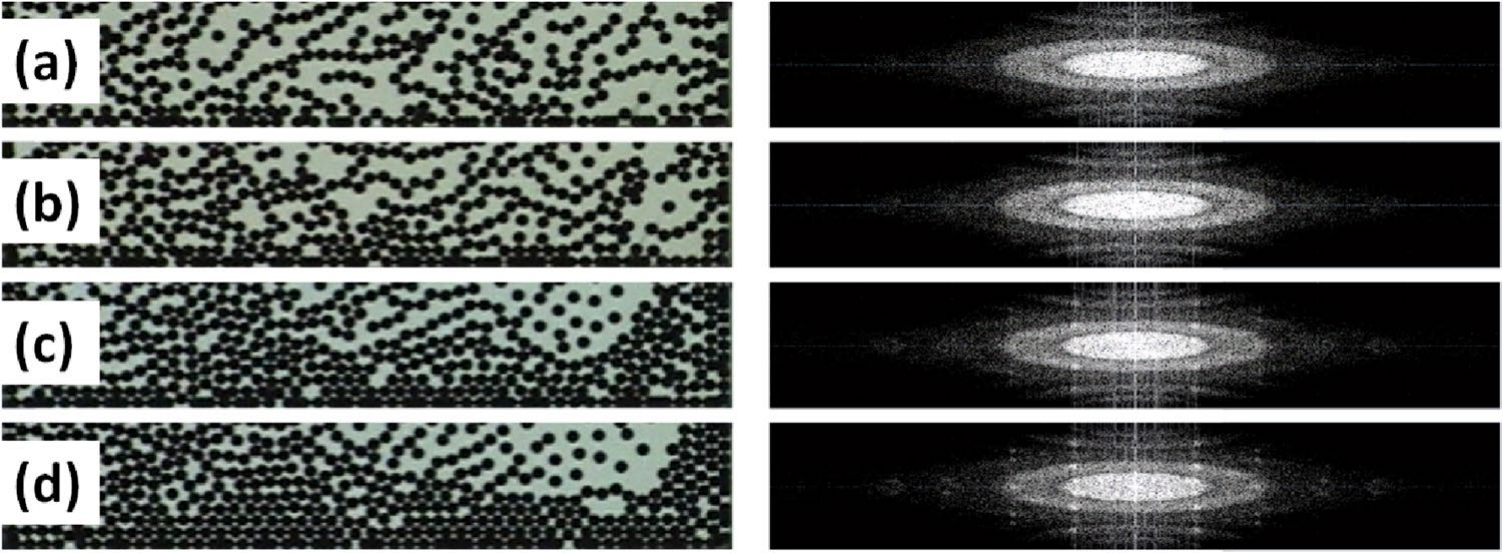
Final particle configurations at different cooling rates and their corresponding fast Fourier transforms. From (**a**) to (**d**), the cooling rates are 1, 0.2, 0.06 and 0.02 G/s. At the lowest cooling rate, crystallization is observed.

Figure 1 shows the final configurations that the system reaches after the system has been quenched at different cooling rates and their corresponding fast Fourier transform. If the rate of cooling is high enough, the formation of disordered structures can be identified as gel-like and glass-like structures. If the rate of cooling is small enough, one observes the formation of crystalline ordered structures. For all cases, one can clearly observe how the collective dynamical arrest phenomenon precedes the formation of solid structures. We discuss the dynamical behaviour of the system using the MSD, denoted by $\langle {\rm{\Delta }}{r}^{2}(t)\rangle$ in the graphs, in the three regions (R1, R2, and R3), for temporal windows at different waiting times *t*_*w*_. From the behaviour of the MSD at each temporal window, we determine the corresponding effective diffusion coefficient. As expected, we observe that the particle concentration gradient yields different dynamical properties in these regions.

**Figure 2:**
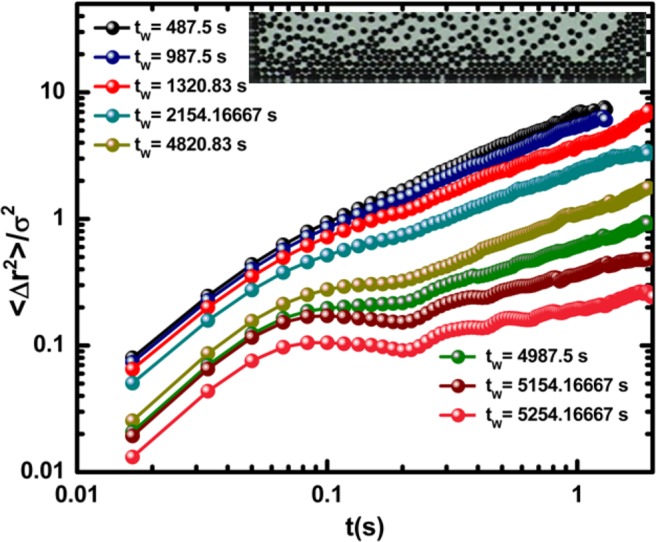
Mean square displacement as a function of time for several temporal windows for the most concentrated region, for the case of the slowest cooling rate. The inset shows a particle configuration 2.5 min after the beginning of the experiment.

In this work, we focus our attention on the crystallization process, namely, in the process of pattern formation that occurs at the lowest cooling rate. Figure 2 shows the MSD behaviour (measured in terms of the particle diameter unit *σ*) in a time window in R1. Here, it is possible to identify the general characteristic trends exhibited by the molecular fluids where solidification is observed. At high temperatures MSD shows a ballistic short segment and then exhibits a diffusive behaviour. As the waiting time increases, the slope of the diffusive segment decreases. Eventually, MSD curves reach a plateau indicating that the particle motion is confined. Thus, the dynamics corresponds to a solid configuration. This behaviour is similar for all higher cooling rate cases. Thus, from the MSD behaviour, one cannot determine whether the reached structure is or is not crystalline. By analysing the behaviour of the effective diffusion coefficient *D* as a function of the effective temperature as the system is cooling down, we are able to calculate the characteristic critical temperature, *T*_*c*_, at which crystalline structures form. We will use this fact to grow a large crystal as described below.

**Figure 3:**
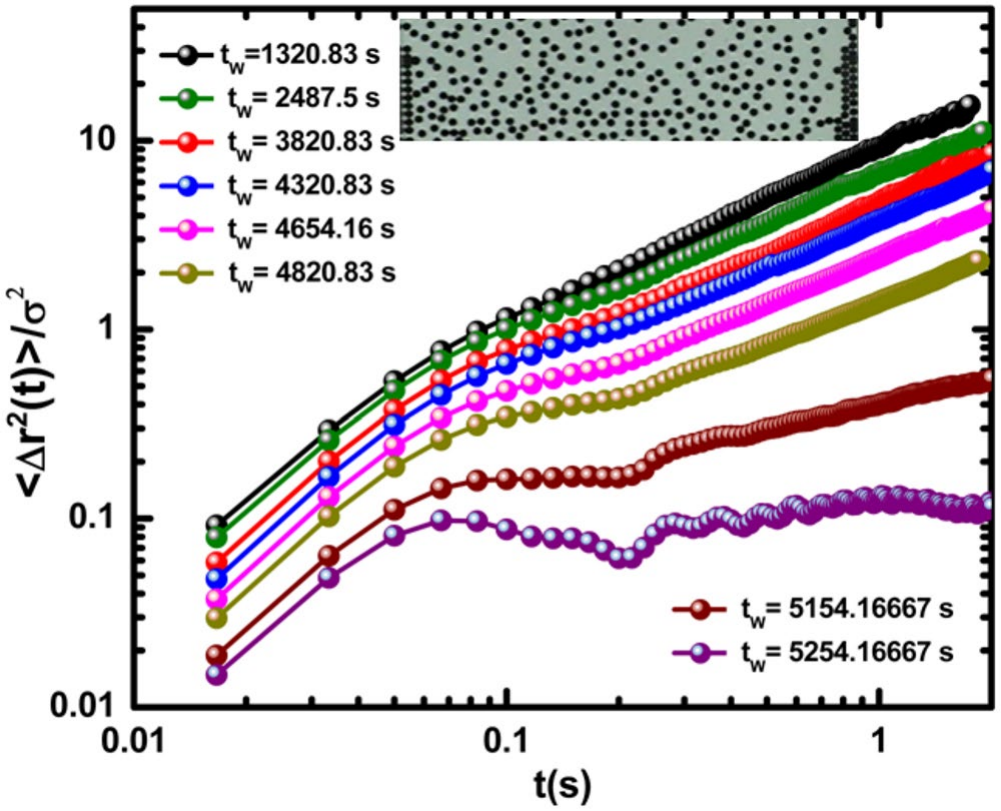
Mean square displacement in the semi-concentrated region. A snapshot of the particle configuration after 2.5 min is shown in the inset of the figure.

Figure 3 shows the MSD curves measured in R2. There, the effective interactions among particles are less strong than in R1. The MSD curves exhibit nonlinear behaviour as a function of time. It is worth mentioning that during the final stages of the experiment, the MSD curves evolved slowly to the curve corresponding to a solid structure. This is a clear indication that the caging phenomenon is also present in the particle dynamics in R2. In this semi-concentrated region, one also observes chain-shaped structures and small clusters.

**Figure 4:**
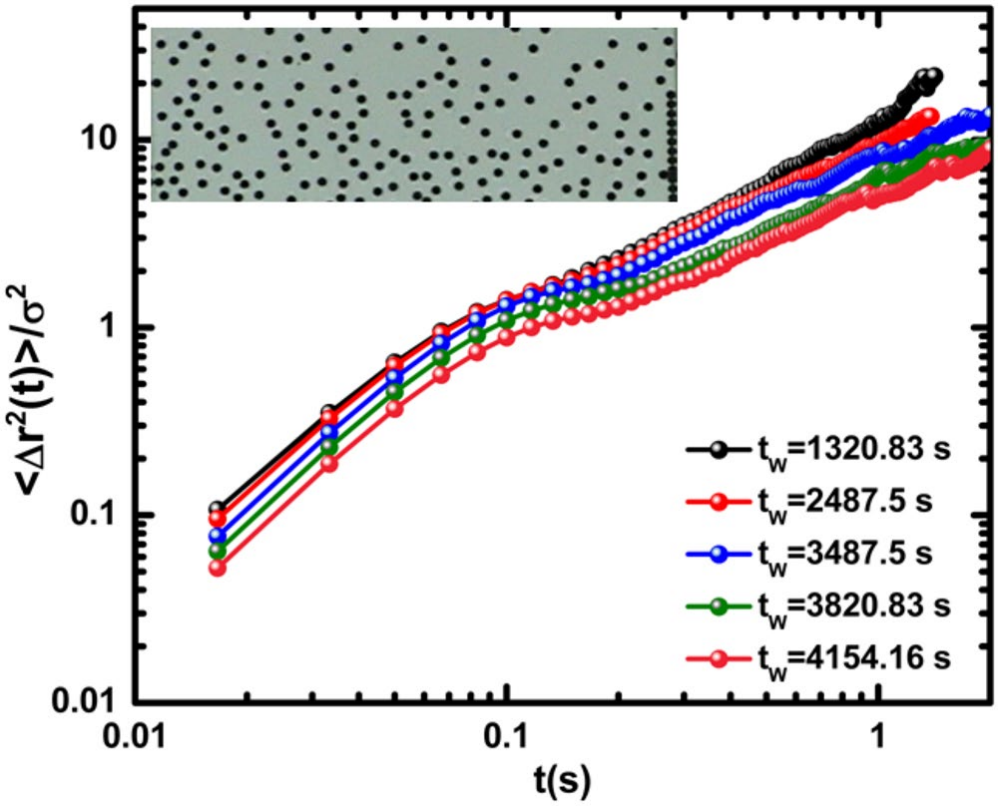
Mean square displacement in the diluted region. The constant increasing behaviour after a long time indicates that the effective diffusion coefficient of the particles is almost constant in R3. Inset: Structure after 2.5 min is shown.

Figure 4 shows the MSD curves for particles in R3. Although this region of the cell is more dilute, the concentration of particles is sufficient for interaction among particles, leading to changes in the MSD behaviour. In these curves, the transition to the solid state is not observed as in the previous cases.

**Figure 5:**
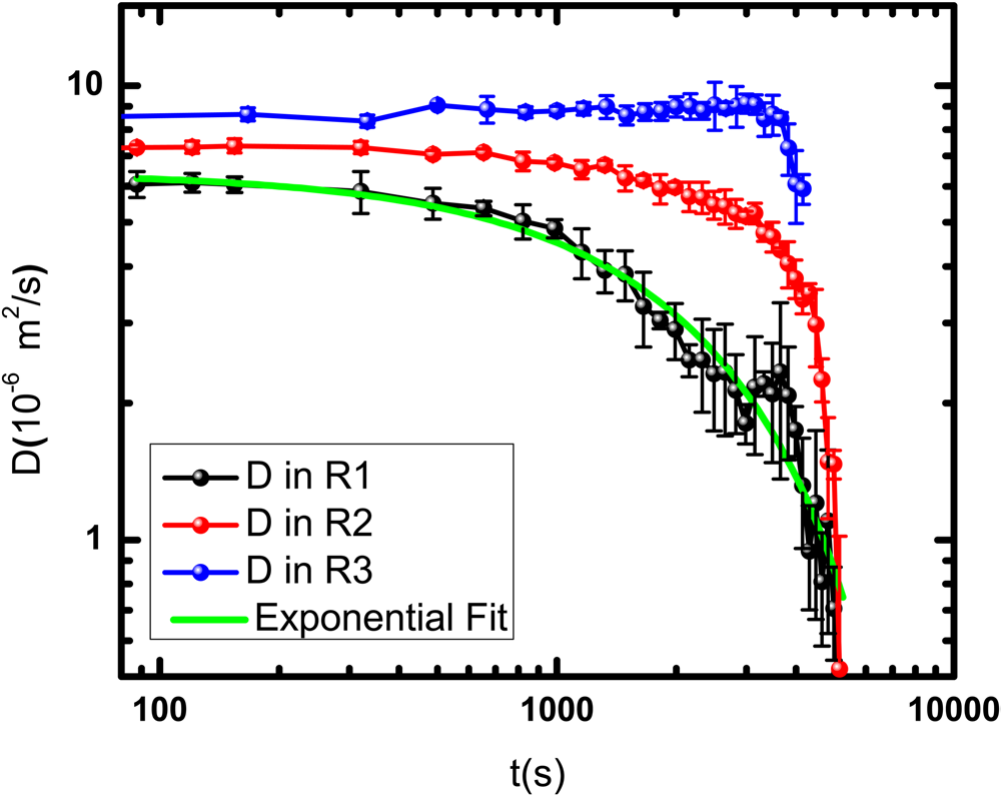
Time evolution of the effective diffusion coefficient in the three regions. A fit to a decreasing exponential function in R1 is shown (green continuous line).

Figure 5 shows the effective diffusion coefficients of the three regions, R1, R2, and R3. One observes that as expected, the diffusion movement of the particles is larger in region R3 than in R2 and R1. Of course, all data in Fig. 5 correspond to the same temporal windows to properly compare the dynamics in the three regions. The effective diffusion coefficient D was obtained by fitting a linear function to the first ten points of each MSD curve using the least squares method, considering both the ballistic part of the curve and the adjacent part of the curve that could be diffusive, subdiffusive or arrested depending on the effective temperature. It is interesting to observe that D in R3 is constant during most of the experimental time. In R2, D experiences small changes. In R1, D decays exponentially.

The dynamic behaviour of the system in the region where crystallization is taking place can be described as follows. Because the particle concentration is grater in R1 than in the other regions, the interaction among the particles is also relatively stronger. As the system is undergoing a cooling process, the transition to a solid state is first observed in this region. While time increases, the effective temperature decreases, and eventually, the system reaches a condition in which the effective temperature is near the critical temperature *T*_*c*_ where crystalline structures start forming. If the system is far from this critical amplitude, crystallization is not possible, and sporadic attempts at nucleation are unstable. Of course, these conditions of effective temperature *T*_*c*_ and its corresponding critical particle concentration *ϕ*_*c*_ determine a critical mean pressure value *P*_*c*_ over the particles near crystalline structure. Given these conditions, particles still have kinetic energy comparable with their potential energy but enough to explore regions of the system beyond their diameter. Particles near a consolidated aggregate collide simultaneously with various aggregated and isolated neighbours. Particles lose kinetic energy without affecting the configuration of the consolidated cluster eventually finding a position of local minimum energy when the particle becomes part of the consolidated aggregate. We will use this fact to grow a large crystal, as we will discuss below.

**Figure 6:**
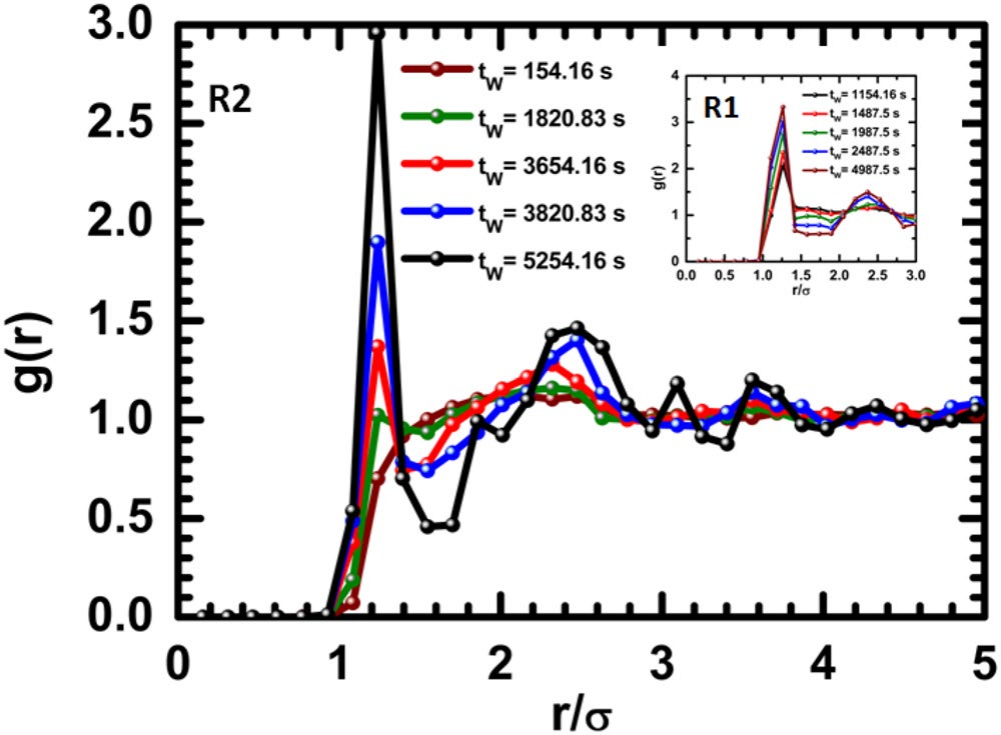
Time evolution of the radial distribution function in the semi-concentrated region R2. Inset: The radial distribution function for several times for the more concentrated region R1. Note that this curve roughly resembles the characteristics of the radial distribution function for a molecular simple liquid.

The time-dependent structural evolution of the system by regions was also analysed. We focus our attention on the structural evolution of the system in R1 and R2, where we find an aggregation of particles. From our measurements using the software package ImageJ, we obtain the radial distribution function and its temporal evolution. Figure 6 shows the radial distribution function in R2 and R1. Our observation of R2 at early times reveals that small clusters of particles start growing, and after some time, a region with local order is formed. This configuration resembles the liquid short order configuration. In R1, the radial distribution function shows the first peak at an early temporal windows; this is because the gradient of the concentration propitiates the particles to dwell closer to each other. The first peak of the radial distribution function appears at a distance slightly larger than *σ*; this means that the particles are almost in contact. As time advances, the first peak becomes higher and a second peak appears. At the last temporal window, the second peak becomes well defined at a position near but slightly farther than 2*σ*. At longer times, particles in R1 become arranged in well-defined solid structures.

**Figure 7:**
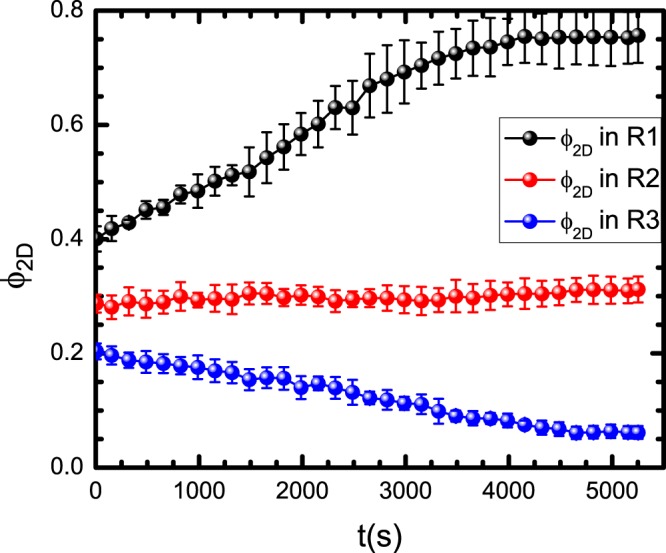
Time evolution of the particle concentration in R1, R2 and R3. In R1 at long times, a plateau emerges. In R2, the particle concentration remains constant, and in R3, the particle concentration decreases.

Now we will discuss how to obtain the critical values of the particle concentration and the effective temperature that promote crystal formation in R1. From previously discussed MSD curves, we learned that at certain quenching conditions, eventually, the granular system goes to an arrested structure. Before this state, particles undergo the caging effect, *ie*., and particle motion is restricted to small displacements around a position. In Fig. 5, the behaviour of the effective diffusion coefficient, D, is shown for different temporal windows. One observes that the value of D decays in an exponential-like behaviour. Considering this, an exponential function is fitted to the experimental data. From this, one may estimate a mean decay time, *t*_*α*_. This is the time at which the effective diffusion coefficient would decay to half of its initial value. We use this mean decay or critical time, *t*_*α*_, to obtain the critical value of the field amplitude *A*_*c*_, of the applied magnetic field. We assume that the proper conditions to grow a crystal are such that the system has a magnetic field applied with amplitude *A*_*c*_ that corresponds to the critical value *T*_*c*_ of the effective temperature. To obtain the corresponding critical particle concentration *ϕ*_*c*_, we now analyse the behaviour of the particle concentration in the different regions. Figure 7 shows the behaviour of the area fraction *ϕ*_2*D*_ occupied by the particles as a function of time in R1, R2, and R3. In R1, *ϕ*_2*D*_ starts at a value of approximately 0.4 and then increases linearly with time. For longer times, starting at approximately 2500 s, although an approximately linear behaviour continues, a change is observed in the slope. A plateau in the graph indicates that particles have formed a solidified region, and the average particle concentration in R1 has achieved its maximum average value, *ϕ*_2*D*_ ≈ 0.75. Clearly, crystallization begins when a critical particle concentration in R1 is reached and when the amplitude of the applied field is tuned to generate the critical temperature value. It is interesting to observe that the particle concentration in R2, the region just above where crystallization is occurring, had the same value approximate value, that is *ϕ*_*c*_ ≈ 0.3. In R3, *ϕ*_2*D*_ decreased over the entire time.

**Figure 8:**
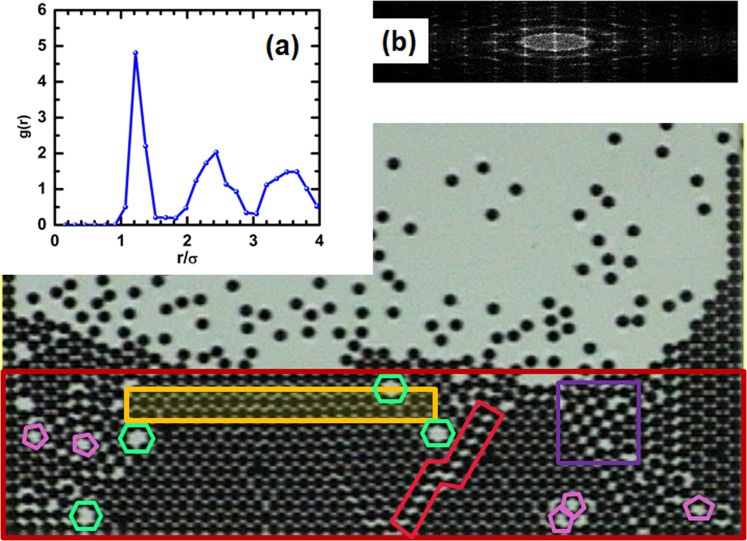
The big picture shows a large crystal is obtained experimentally when the particle concentration is kept constant in the non-consolidated region, and the effective temperature is kept at its critical value. A dislocation is marked in red. A squared lattice region is marked in purple. Hexagonal vacancies are marked in green, and pentagonal vacancies are marked in pink. (**a**) is the radial distribution function for the crystal, and (**b**) is the corresponding fast Fourier transform.

We expect that if the delicate balance of these critical conditions of *T*_*c*_ and *ϕ*_*c*_ is held during the crystal growing process, one should be able to grow large crystals. Furthermore, to contribute to maintaining this delicate balance, the number of particles in the system must be kept constant by adding particles to the system in the non-consolidated region R3 at the same ratio such that they become attached to the solid structure in R1. Figure 8 shows a large crystal grown by keeping the effective temperature constant at its critical particle concentration value and adding particles into the non-consolidated region to propitiate a constant feeding to the crystal growth. Supplementary Movies 1–3 show different stages of the crystal growth shown in Fig. 8. Most of R1 is covered by a crystalline hexagonal lattice. Nevertheless, some other structures are present as well. These structures include a rhombohedral structure, a region of a square lattice, and even a small region with a pentagonal structure. The structure also exhibits several crystalline defects. One may observe vacancies and dislocations. Notice that in contrast to previously reported results regarding the formation of ordered structures in some related granular systems^22^, in this case, crystallization is not assisted by commensurability in the confinement. In our system, crystallization is a collective phenomenon preceded by the capture of particles by a local minimum of energy. Subsequently, these particles lose energy colliding with their neighbours under dynamic arrest conditions. In Supplementary Movies 4–6, the behaviour of individual particles in a short time in the three different particle concentration regions can be observed.

**Figure 9:**
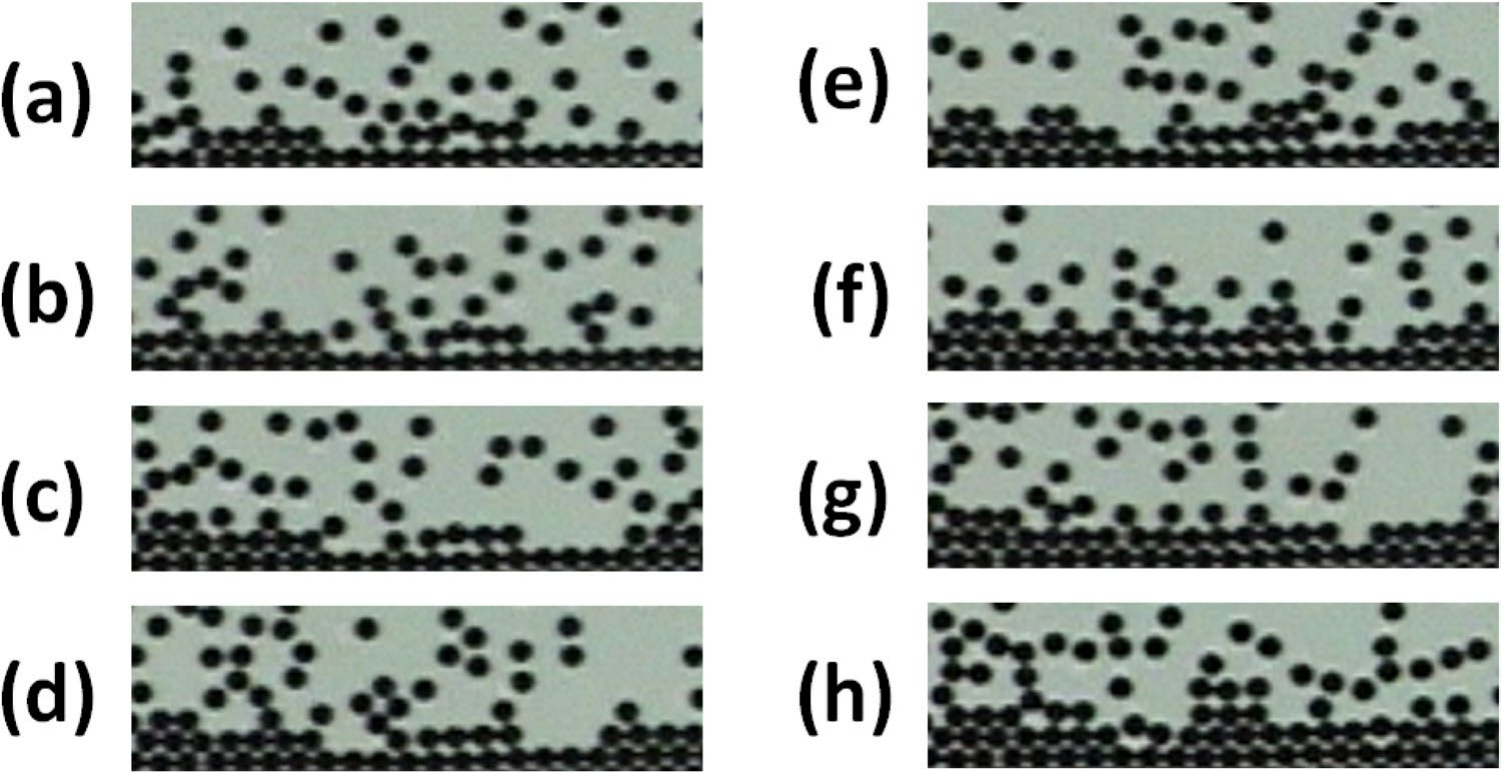
Sequence of aggregation in an early stage of crystal formation. The region is the yellow rectangle marked in Fig. 8. Note that particles are initially in disordered locations and are then an early stage of crystal formation. This region evolves to a more ordered configuration.

A sequence of images of the formation of a part of a crystal is shown in Fig. 9. We have observed that some particles could adhere at a site corresponding to a local minimum of energy but not necessarily the greatest minimum. The interactions with the surrounding aggregated particles and the collisions with those non-aggregated particles, make these particles move and allocate to other positions until they find the lowest energy configuration. In understanding this particular process and to obtain some insights on some other similar situations, this system demonstrates how important the collective interactions are to the solidification processes.

## Remarks

In this work, we report on a study of the static and dynamic structural properties of a granular 2D system that is formed by a large set of steel magnetic spherical beads and is under the action of an external variable magnetic field. The system model the phenomena that occur in the processes of quenching in a molecular fluid. However, in this granular system, these phenomena can be observed in detail by the naked eye. We focused our attention on the crystallization process. The situation analogous to the cooling down process, in this case, is generated by the reduction of the applied field’s amplitude. We were able to find the conditions in which it is possible to observe the nucleation process and the ulterior crystal growth. One of the important facts we prove here experimentally is that in the absence of confinement, crystallization of particles with a repulsive short-range interaction is possible. These systems allow us to study in detail the processes of dynamical arrest that precede the aggregation that yields solid-like structures, from disordered glassy structures to ordered crystalline structures. We found the critical particle concentration and effective temperature threshold conditions at which the system crystallizes. Based on these results, we proposed a procedure that allowed us to grow a large crystalline structure. In this large crystal, we can observe the formation of different defects. The experimental arrangement is very simple, versatile, and can be easily modified or adapted to investigate many other phenomena related to crystal growth or the stability of different packing structures^25^.

## Methods

**Figure 10:**
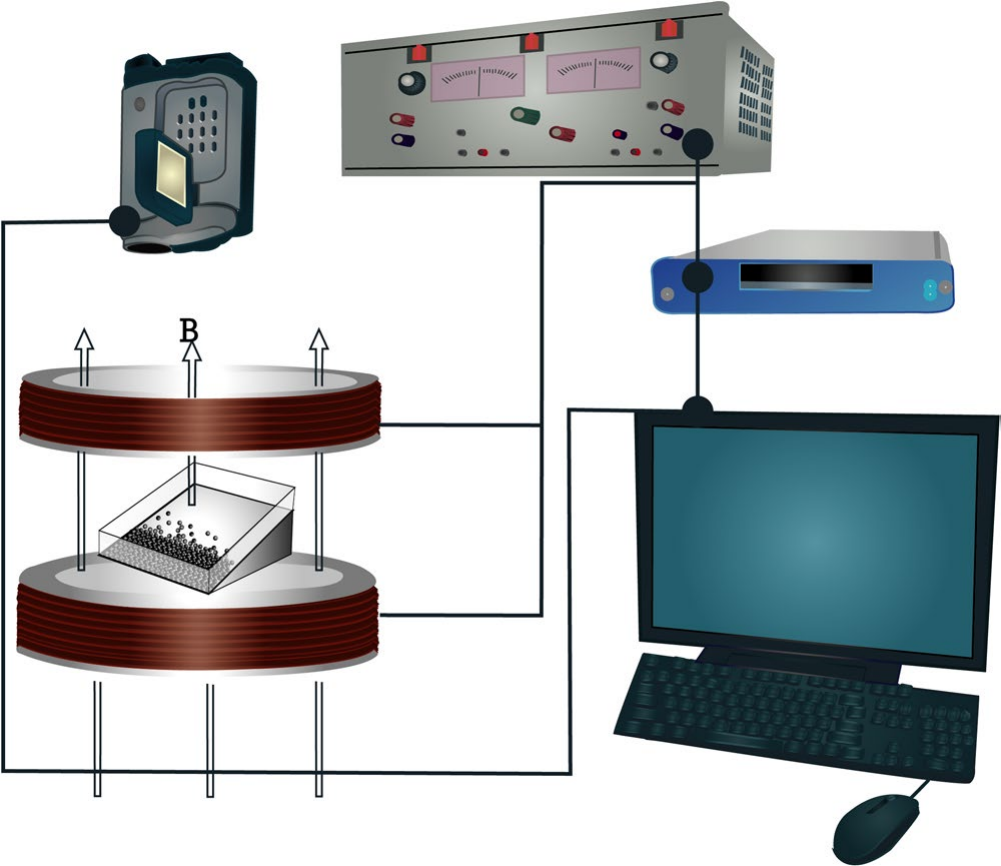
Experimental setup. The tilted cell is in the middle of the Helmholtz coils where the magnetic field is homogeneous. The controlling signal is sent from a PC to a power amplifier through a DAQ card.

**Figure 11:**
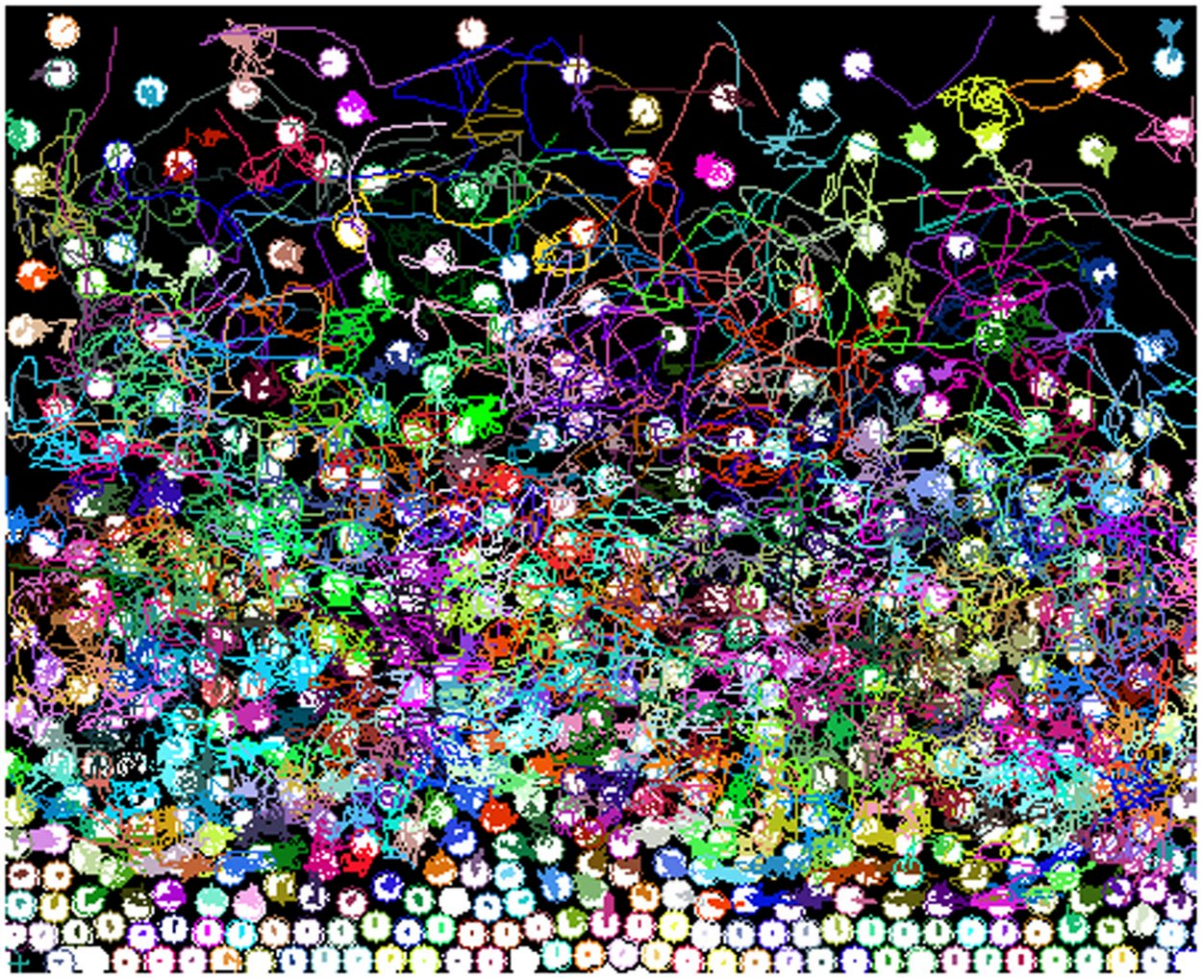
Typical trajectories in a sample. The coexistence of diffusive trajectories and confined trajectories is observed while a crystal is growing.

Our system consists of a bed of magnetized spherical steel beads with diameter *σ* = 1 mm deposited on a rectangular glass plate confined by acrylic walls. The plane is slightly tilted to generate a particle concentration gradient. Figure 10 shows a schematic diagram of the experimental setup. The motion of the beads is induced by the application of an external, time-dependent, magnetic field^15^. The magnetic field is produced by pairs of Helmholtz coils fed by a power amplifier and driven by a function generator. Gravity ensures that the particles always remain on the surface of the cell, making the particle distribution behaviours an authentic 2D system. It is possible to follow the motion of individual particles in this system by using standard optical videomicroscopy. A CCD video camera was used to record the dynamics of the system. In the fast quenching, the video recording took approximately 90 s, and in the experiment with the slowest quenching, the recording was around of 5500 s. The videos were recorded at a standard resolution of 720 × 480 pixels at 30 fps in AVI interlaced format. We analysed both, the odd and even fields of the image separately to increase the time resolution to *τ* = 1/60 s. Particle positions and trajectories were obtained using ImageJ and its plugin Mosaic^26^. Figure 11 shows typical trajectories in a growing crystal.

The applied magnetic field *B* is given by the expression

1 $$B={B}_{c}+{B}_{o}\,\sin (2\pi ft),$$

where *B*_*c*_ = 62.8*G* is a constant magnetic field, *B*_*o*_ and *f* are the amplitude and the frequency of the unsteady component of the applied field, *B*_*o*_ takes values in the range 0*G* to 71.9*G*, and *f* = 9.25 *Hz* is kept fixed.

Briefly, the origin of the particles’ random motion is as follows: when a particle, with a permanent magnetic moment, is acted on by an applied magnetic field, to minimize the magnetic energy, the particle rotates trying to align its magnetic moment with the applied field. Because the magnetic field is not static but sinusoidal, the magnetic field eventually changes direction. While a particle rotates following the field direction, it rolls on the cell surface due to the friction with the surface of the container; thus, the particle moves in a linear trajectory. The direction in which this particle rolls is random because a sphere has a neutral equilibrium. Eventually, the particle obtains into a position where it will again change direction into a new unpredictable direction. Additionally, as a result of this movement, when two particles approach each other, these particles suffer a repulsive interaction because the constant contribution of the magnetic field *B*_*c*_ produces a repulsive interaction between particles. Thus, the dynamics could be very complex depending on parameters, such as the particle concentration and the intensity and frequency of the applied magnetic field.

We experimentally analised the motion of the magnetic particles subjected to different cooling rates for a given particle concentration gradient, which is generated by the slight tilt of the cell surface. Different quenching conditions of quenching led to different structural configurations. The analysis of the dynamics and structure of the system was made by dividing the observation area into three regions with different particle concentrations.

## Supplementary information

Supplementary Movie 1

Supplementary Movie 2

Supplementary Movie 3

Supplementary Movie 4

Supplementary Movie 5

Supplementary Movie 6

## References

[1] Sánchez-Daz LE, Ramrez-González P, Medina-Noyola M (2013). Equilibration and aging of dense soft-sphere glass-forming liquids. Physical Review E.

[2] Heneghan AF, Haymeta ADJ (2002). Liquid-to-crystal nucleation: A new generation lag-time apparatus. Journal of Chemical Physics.

[3] Bergfors T (2003). Seeds to crystals. J. Struct. Biol..

[4] Yau ST, Vekilov PG (2000). Quasi-planar nucleus structure in apoferritin crystallization. Nature.

[5] Oxtoby DW (2000). Catching crystals at birth. Nature.

[6] Anderson VJ, Lekkerkerker HNW (2002). Insights into phase transition kinetics from colloid science. Nature.

[7] Lu PJ (2008). Gelation of particles with short-range attraction. Nature.

[8] Adams DJ, Mullen LM, Berta M, Chen L, Frith WJ (2010). Relationship between molecular structure, gelation behaviour and gel properties of fmoc-dipeptides. Soft Matter.

[9] Hunter GL, Weeks ER (2012). The physics of the colloidal glass transition. Reports on Progress in Physics.

[10] Weeks ER, Crocker JC, Weitz DA (2007). Short- and long-range correlated motion observed in colloidal glasses and liquids. Journal of Physics: Condensed Matter.

[11] Weeks ER, Weitz DA (2002). Properties of cage rearrangements observed near the colloidal glass transition. Physical Review Letters.

[12] Kozina A, Daz-Leyva P, Palberge T, Bartsch E (2014). Crystallization kinetics of colloidal binary mixtures with depletion attraction. Soft Matter.

[13] Prileszky TA, Furst EM (2016). Crystallization kinetics of partially crystalline emulsion droplets in a microfluidic device. langmuir.

[14] Shi H-H (2017). Progress of crystallization in microfluidic devices. Lab on a Chip.

[15] Tapia-Ignacio C, Garcia-Serrano J, Donado F (2016). Nonvibrating granular model for a glass-forming liquid: Equilibration and aging. Physical Review E.

[16] Donado F, Sausedo-Solorio JM, Moctezuma RE (2017). Dynamical and structural properties of a granular model for a magnetorheological fluid. Physical Review E.

[17] Donado F, Moctezuma RE, López-Flores L, Medina-Noyola M, Arauz-Lara JL (2017). Brownian motion in non-equilibrium systems and the ornstein-uhlenbeck stochastic process. Scientific Reports.

[18] Moctezuma RE, Arauz-Lara JL, Donado F (2018). Structural characterization of a magnetic granular system under a time-dependent magnetic field: Voronoi tessellation and multifractal analysis. Physica A.

[19] Reis PM, Ingale RA, Shattuck MD (2007). Caging dynamics in a granular fluid. Physical Review Letters.

[20] Rietz F, Radin C, Swinney HL, Schröter M (2018). Nucleation in sheared granular matter. Physical Review Letters.

[21] Zhao S-C, Sidle S, Swinney HL, Schröter M (2012). Correlation between voronoi volumes in disc packings. Europhysics Letters.

[22] Carvente O, Ruiz-Suárez J (2005). Crystallization of confined non-brownian spheres by vibrational annealing. Physical Review Letters.

[23] Panaitescu A, Reddy KA, Kudrolli A (2012). Nucleation and crystal growth in sheared granular sphere packings. Physical Review Letters.

[24] Morales-Barrera DA, Rodríguez-Gattorno G, Carvente O (2018). Reversible self-assembly (fcc-bct) crystallization of confined granular spheres via a shear dimensionality mechanism. Physical Review Letters.

[25] Berryman JG (1983). Random close packing of hard spheres and disks. Physical Review A.

[26] Sbalzarini IF, Koumoutsakos P (2005). Feature point tracking and trajectory analysis for video imaging in cell biology. J Struct Biol.

